# Benthic pH gradients across a range of shelf sea sediment types linked to sediment characteristics and seasonal variability

**DOI:** 10.1007/s10533-017-0323-z

**Published:** 2017-03-31

**Authors:** B. Silburn, S. Kröger, E. R. Parker, D. B. Sivyer, N. Hicks, C. F. Powell, M. Johnson, N. Greenwood

**Affiliations:** 1 Centre for Environment Fisheries and Aquaculture Science, Pakefield Road, Lowestoft, Suffolk NR33 0HT UK; 2 Scottish Marine Institute, Oban, Argyll PA37 1QA UK; 3 University of East Anglia, Norwich Research Park, Norwich, Norfolk NR4 7TJ UK

**Keywords:** Sediment pH, Oxygen, Carbon cycle, Microelectrode profiles, Celtic sea, Shelf sea biogeochemistry

## Abstract

This study used microelectrodes to record pH profiles in fresh shelf sea sediment cores collected across a range of different sediment types within the Celtic Sea. Spatial and temporal variability was captured during repeated measurements in 2014 and 2015. Concurrently recorded oxygen microelectrode profiles and other sedimentary parameters provide a detailed context for interpretation of the pH data. Clear differences in profiles were observed between sediment type, location and season. Notably, very steep pH gradients exist within the surface sediments (10–20 mm), where decreases greater than 0.5 pH units were observed. Steep gradients were particularly apparent in fine cohesive sediments, less so in permeable sandier matrices. We hypothesise that the gradients are likely caused by aerobic organic matter respiration close to the sediment–water interface or oxidation of reduced species at the base of the oxic zone (NH_4_^+^, Mn^2+^, Fe^2+^, S^−^). Statistical analysis suggests the variability in the depth of the pH minima is controlled spatially by the oxygen penetration depth, and seasonally by the input and remineralisation of deposited organic phytodetritus. Below the pH minima the observed pH remained consistently low to maximum electrode penetration (ca. 60 mm), indicating an absence of sub-oxic processes generating H^+^ or balanced removal processes within this layer. Thus, a climatology of sediment surface porewater pH is provided against which to examine biogeochemical processes. This enhances our understanding of benthic pH processes, particularly in the context of human impacts, seabed integrity, and future climate changes, providing vital information for modelling benthic response under future climate scenarios.

## Introduction

Continental shelf seas cover approximately 7% of the total ocean surface area, but have a proportionally greater role in ocean productivity than the open ocean (Middelburg and Soetaert 2005) and are at greater risk from human impacts (Halpern et al. 2015). International concern over increasing carbon dioxide concentrations in the atmosphere, and associated acidification of seawater, has led to an increase in studies focusing on water column pH (Doney et al. 2009). In contrast, benthic pH measurements are still very rare due methodological and cost constraints. A suite of biogeochemical processes influences pH values in marine sediments, increasing or decreasing free proton concentrations in porewaters. These processes are mainly linked to cycles of carbon, oxygen, nitrogen, phosphate, silicate, sulphur, iron and manganese and are associated with processes such as heterotrophic respiration, chemoautotrophic activity, photosynthesis, precipitation, and dissolution of calcium carbonate. These processes have a profound effect on the overall pH balance in marine sediments (Cai et al. 1995; Reimers et al. 1996; Revsbech et al. 1983; Soetaert et al. 2007; Stahl et al. 2006; Wenzhöfer et al. 2001). Due to over 30 processes, which can alter the porewater H^+^ concentrations, it remains challenging to interpret observed variability and derive both correlative and causative links to other biogeochemical parameters. In addition, many of these reactions are redox sensitive or linked to reduction or reoxidation cycles. Sediment oxygen profiles measurements on the other hand have become more commonplace (Glud 2008; Rabouille et al. 2003), although they are still rarely acquired within routine monitoring programmes. From such profiles the oxygen penetration depth can be determined. Oxygen penetration depth (OPD) as described by Rabouille et al. (2003) is the depth where O_2_ microelectrode signal reached the zero current and is the limit of measurable free dissolved oxygen, also known as the oxic zone. This depth is critical in controlling subsequent CO_2_ generation pathways or H^+^ generating cycles linked to organic matter degradation which occur, theoretically, once oxygen is consumed and/or deeper in the sediment. For example, those cycles linked to N, S, Fe and Mn reduction and oxidation processes and controlled by the oxic-suboxic-anoxic boundary with depth (Cai and Sayles 1996; Canfield et al. 1993).

**Fig. 1:**
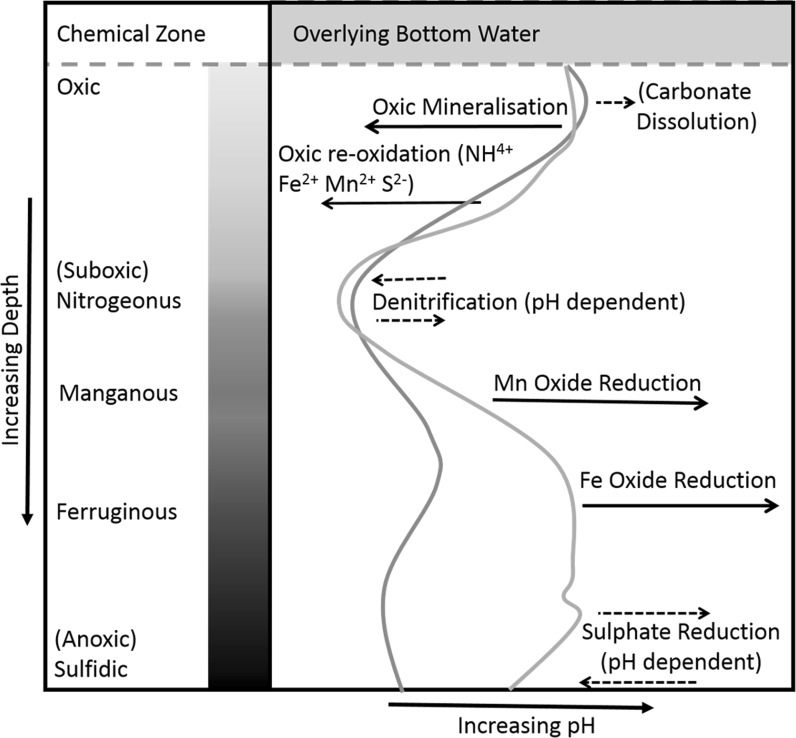
Exemplar measured sediment pH profiles (*green* and *orange lines*) for diffusive (muddy sand) surficial sediments showing the main chemical processes that drive changes in pH. Those processes that have a strong influence on pH are shown with a *solid black arrow*, the *broken arrows* are used to denote those processes that have a weaker, or more uncertain, influence. Chemical zones within the sediment are described using Canfield and Thamdrup (2009). Example profiles were collected in North Sea sediments as part of the Defra Seabed Integrity–ME5301 (Defra 2013) and supported by the UK Ocean Acidification programme and the Cefas Seedcorn programme. (Colour figure online)

Several biogeochemical processes that drive changes in pH are known to occur within surficial sediments (Middelburg and Levin 2009; Canfield and Thamdrup 2009). An idealised picture of how pH profiles are affected by some of the more important processes are shown in Fig. 1. This uses the geochemical zones from Canfield and Thamdrup (2009), along with some of the major processes presented in Soetaert et al. (2007), to annotate idealised pH profiles that are based on pH measurements taken from the central North Sea (Defra Seabed Integrity—ME5301; Cefas survey CEND11/11) using the same sampling method as described in this paper. The original pH profiles (shown in green and orange) were measured to a depth of approximately 3 cm in cores of a diffusive sandy mud with low carbonate content, but relatively high iron and manganese content. The pH ranged from 6.6 to 7.6. As the transport of solutes through this type of sediment is dominated by diffusion, the pH steeply decreases with depth in the oxic layer as a result of the oxic respiration of organic matter and the reoxidation of reduced species (Mn^2+^, Fe^2+^, NH4^+^ or HS^−^) (Cai and Reimers 1993). By contrast, a sediment dominated by advective transport would have a relatively flat pH profile, even though it would have an enhanced rate of oxic mineralisation (Janssen et al. 2005; Bühring et al. 2006). This is due to the increased flushing of oxygen rich water from the overlying water column through the sediment, creating rapid equilibration of chemistry between the sediment in the upper layers and the water column. Variability in a pH profile is, therefore, affected by temporal or spatial changes in site specific conditions. In particular, the grain size distribution, and permeability, which has a large effect on the depth and mechanism of transport of solutes in the sediment (Huettel and Rusch 2000; Huettel et al. 2003; Precht and Huettel 2003; Precht et al. 2004). Grain size also affects other parameters that are important for setting the geochemical zonations related to the processes listed above, such as organic carbon distribution, redox state and bioturbation (Huettel and Webster 2001; Volkenborn et al. 2007). The interplay between these sediment processes can lead to contrasting pH profile shapes and overall pH levels. It is worth noting that oxic mineralisation is initially very important in reducing pH and controlling pH near the sediment surface, because it produces CO_2_. This in turn dissociates to produce [H^+^] ions. However, the dissociation reaction of CO_2_ in water is a reversible chemical equilibrium which is itself pH dependent (Libes 2009). Thus at lower pH (Soetaert et al. 2007) this process becomes less important and further reduction in pH within the oxic parts of the sediment is driven by reoxidation reactions, notably the reoxidation of Mn^2+^, Fe^2+^ and FeS or HS^−^ by oxygen. In some sediments, this may cause more intense pH minima than the pCO_2_ changes itself (Cai and Reimers 1993). Below the OPD, organic matter mineralization by microbial activity involves multiple redox reactions, following the order of Gibbs free energy (Berner 1980; Boudreau 1992), which drive nitrogen, sulphur, and iron and manganese cycles and associated redox processes. Whilst all marine carbon remineralisation reactions liberate CO_2_, the nature of the CO_2_ dissociation reaction means that the effect of these reactions at any depth given is largely pH dependent. Additionally, denitrification and sulphate reduction also consume nitrate and sulphate respectively, so that the effect of these two processes is less pronounced than with oxic mineralisation (Soetaert et al. 2007). By contrast, iron and manganese reduction, which occurs post denitrification, both consume far more protons than might be produced by the CO_2_ evolved from either reaction, therefore both of these reactions act to increase the pH (Koschorreck et al. 2003). The potential depth and directional effects of these main pH affecting carbon remineralisation and redox reactions are annotated in Fig. 1.

Considering these main processes, some minima in pH could be expected to occur close to the OPD in cohesive sediments, where oxic respiration and reoxidation of reduced species diffusing from below occurs, but unconnected to the source of buffered seawater from the overlying water column. However, higher permeability sediments, which are dominated by active porewater flow and hence differing redox, organic carbon contents and state, may differ in their iron, manganese or carbonate concentrations. This would result in different pH profiles. Additionally, sediments rarely behave in a ‘textbook’ manner with a perfect cascade of reduction reactions as they are rarely temporally or spatially homogenous, so chemical zones may be present or absent depending on the biogeochemical composition (Canfield and Thamdrup 2009) or heterogeneity introduced by patchiness in biological processes/mixing or organic matter deposition/mixing. For example, the presence of benthic macrofauna and their differing feeding modes can lead to the boundaries between biogeochemical zones becoming less distinct (Zhu et al. 2006) or micro-zonation of redox processes within micro-niches (Stockdale et al. 2009) due to the redistributions of organic matter or oxygen. Various types of physical impacts, natural or anthropogenic, can also temporarily or repeatedly disturb sediment biogeochemical zonation, for example storm events, trawling or dredge aggregation/disposal. Therefore, pH profiles reflect a balance between biogeochemical reaction zones and physical and biological sediment reworking.

Given the close coupling of benthic and pelagic processes through diffusion, advection or biota-mediated exchanges, pH distribution in sediments is of significance (Widdicombe et al. 2011) as it provides information about likely net direction of proton exchange across either the sediment:water OR oxic:anoxic interfaces. Both interfaces are indicative of regions of different biogeochemical processes and coupled CO_2_ production. Furthermore, the recorded pH profiles clearly illustrate the natural pH variability experienced by benthic epifauna and infauna. The pH variability is of great relevance to climate change motivated experiments, where benthic organisms are exposed to predicted future pH water column ranges, as well as changes in other indicators such as partial pressure of carbon dioxide (*p*CO_2_) [a measure of the degree of saturation of the sample with CO2 gas (Riebesell et al. 2010)]. Changes in sediment pH through ocean acidification could potentially impact benthic dwelling organisms by affecting their biological processes, such as growth, respiration, calcification, metabolic rate and activity (Widdicombe and Spicer 2008). This could impact on biodiversity and, in turn, cycles of carbon and nitrogen within the sediment. Ascertaining a baseline for sedimentary pH will constrain present day ambient conditions and potential drivers, and therefore allow detection of change in the future in response to the increasing pCO_2_.

In this large scale observational field study, microelectrodes were used to record pH profiles in cores taken from surface sediments across a range of sediment types. Concurrently recorded oxygen microelectrode profiles and other sedimentary parameters provide a detailed context in which to interpret the pH data. The presented work is built on the hypothesis that benthic pH profiles are far from uniform, but indeed influenced by and reflective of numerous sediment characteristics and processes with a strong seasonality. Relevant determinates are expected to encompass physical parameters (grain size), closely linked chemical gradients, including carbon input and remineralisation processes, and oxygen penetration depth.

## Methods

### Study area

**Fig. 2:**
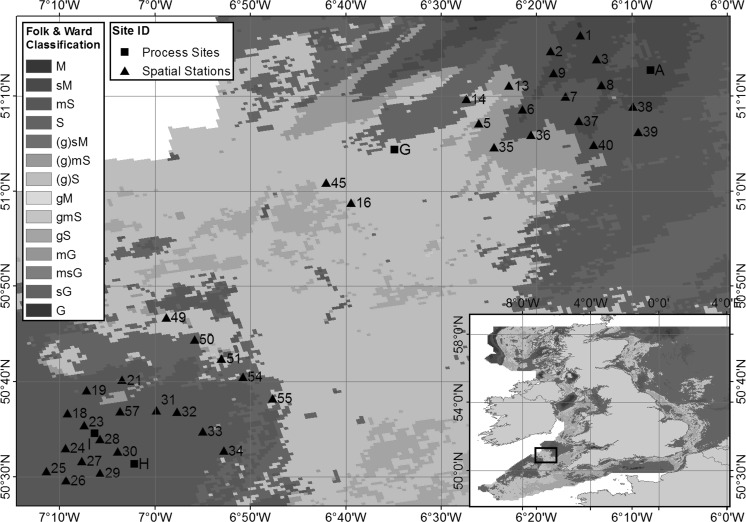
Predicted surficial sediment Folk and Ward textural classifications based on BGS surface sediment maps, with process sites and spatial stations indicated (Stephens 2015; Stephens and Diesing 2015; Folk 1954; Folk and War 1957; Thompson et al. 2017)

Our study focuses on the Celtic Sea region of the north-west European continental shelf. More detail of the study area selection can be found in Thompson et al. (2017). In summary, the area is of consistent depth, averaging 95 m below chart datum and encompasses a wide variety of sediment types (Fig. 2), predominantly muddy sands (24%), sands (13%), slightly gravelly sands (43%) and gravelly sands (12%) (Thompson et al. 2017). Four process sites were chosen (Site A: mud; Site G: sand; Site H: muddy sand; and Site I: sandy mud) for the temporal study and were visited four times (April 2014, March 2015, May 2015 and August 2015). Different sampling time points allowed seasonally variability in biogeochemical parameters to be captured, such as oxygen penetration depth and organic carbon input, influenced by the seasonal bloom conditions (Hicks et al. 2017). The process sites represented end-members of the regional sediment biogeochemical drivers (diffusive (mud); advective (sand)), with two intermediate points (muddy sand and sandy mud). The wider distribution of spatial stations across the targeted survey region was designed to encompass samples from all the regions differing sediment types, particularly ensuring a wide range of percentage fines content. These were sampled during March 2015 (pre-spring bloom) and will put the process sites in context with the wider regional sediment biogeochemistry.

### Sample collection

Sediment samples were collected using a NIOZ box corer with a 300 mm diameter cylindrical barrel. The NIOZ corer produces relatively undisturbed core samples, sealed from below and above to retain porewater structure and overlying bottom water. From this a 100 mm diameter sub core was collected, retaining 10–15 cm of the overlying water, for immediate pH and oxygen microelectrode profiling to avoid sample degradation (Lohse et al. 1996; Sciberras et al. 2016; Teal et al. 2010). In addition, a 50 mm diameter sub-core was collected for Particle Size Analysis (PSA) and particulate organic carbon and nitrogen (OCN), as well as a 60 ml syringe for porosity. These sub-cores were then sliced at 5 cm intervals to a maximum depth of 10 cm.

### Profile acquisition

Oxygen and pH sediment porewater profiles (adjusted to the seawater scale, as described by Cai et al. 1995) were acquired in the same core using a dual mounted motorised micromanipulator profiling head, manufactured by Unisense and connected to a laptop. A Unisense pH-500 microelectrode (Kühl and Revsbech 2001; Revsbech and Jørgensen 1986) was mounted beside an OX-500 microsensor (Revsbech 1989) at a distance of 1 cm apart, with the tips of the probes (500 µm diameter) in the overlying water 2–5 mm above the sediment surface, as determined by eye. An aeration stone was placed at the surface of the overlying water to maintain oxygen concentration. The microelectrodes were deployed at 1 mm depth intervals using a motorised profiling head (Unisense micromanipulator) and linked control software (Sensor Trace Pro v 3.0.6). The electrode was allowed to equilibrate for 15 s at each depth interval before measuring for 3 s. Profiling occurred to a maximum depth of 6 cm. All probes were calibrated daily for result consistency. The OX-500 were calibrated with a 2-point method, using aerated sea water collected from the continuous flow (filtered surface seawater) for 100% saturation and a Sodium Hydroxide and Ascorbic solution for 0% saturation. The pH-500 were calibrated with a 3-point method, using three laboratory standard buffers at pH 4, 7 and 10 (DY021) or 4, 6 and 8 (DY030 and DY034). All pH profiles were then offset for the seawater scale (Cai et al. 1995) using a pH TRIS buffer prepared in artificial seawater (Certified Reference Material, Nemzer and Dickson 2005). All calibrations and measurements were done in the wet-lab at ambient air temperature, however, immediate profiling following recovery reduced the risk of core warming. In some cases, small adjustments were made post-profiling to the depth of the profiled points in order to align the sediment–water interface to 0 cm. Oxygen profiles were then used to determine the OPD (for full details on the regional oxygen dynamics, see Hicks et al. 2017). This was defined as the final depth at which oxygen is measurable by the electrode, as described above, and where the O_2_ microelectrode signal reached a zero in comparison to a Sodium Hydroxide and Ascorbate solution zero (Rabouille et al. 2003). The depth below the surface at which pH first reached a minimum value before increasing again, or first reached an equilibrium/plateau, was recorded as the initial sub-surface pH minima.

### Sediment sample analysis

Sediment slices for PSA were analysed following the National Marine Biological Analytical Quality Control (NMBAQC) method, whereby sediment is split at 0.5 phi (ϕ) intervals, combining laser diffraction of the <1 mm with sieved weights of the >1 mm (Mason 2011). Statistical data and Folk and Ward classifications (Folk 1954; Folk and Ward 1957) were then acquired using the software tool Gradistat (Version 8) (Blott and Pye 2001). Porosity samples were weighed, freeze dried and weighed again to get the dry:wet sediment weight ratio (Danielson and Sutherland 1986). Additional sediment was freeze dried, ground and weighed, before sulphurous acid was added to remove any inorganic carbon. Acidified samples were oven dried at 40 °C, then analysed for OCN using a Carlo Erba EA1108 Elemental analyser (Kirsten 1979).

## Results

Biogeochemical observations were carried out at the Process Sites during the four cruises, providing comparable data for differing seasonal states. During the study site selection process (April 2014) pH profiles were not collected. A summary of the collected parameters is shown in Table 1. Full data sets of sediment characterisation and microelectrode profiles are available from the British Oceanographic Data Centre (http://www.bodc.ac.uk) (Silburn et al. 2017a, b).

**Table 1:** Temporal sediment biogeochemical parameters collected at the process sites during four cruises between 2014 and 2015 at differing points through the spring bloom: DY008 pre-spring bloom (April 2014); DY021 pre-spring bloom (March 2015); DY030 bloom (May 2015); DY034 post-spring bloom (August 2015)

Process site	Cruise	Month	Gravel (%)	Sand (%)	Silt/clay (%)	Oxygen penetration depth (cm)	Sub-surface pH minima depth (cm)	Total organic carbon (%m/m)	Nitrogen (%m/m)	Porosity
Site G	DY008	April 2014	14.26	80.94	4.79	1.4	NA	0.11	0.02	0.44
	DY021	March 2015	0.98	94.68	4.34	5.0	4.3	0.12	0.05	0.38
	DY030	May 2015	0.15	96.98	2.87	1.4	1.2	0.15	0.05	0.39
	DY034	August 2015	0.35	60.25	39.40	0.5	1.4	0.49	0.12	0.58
Site H	DY008	April 2014	0.11	79.65	20.24	1.5	NA	0.31	0.05	0.48
	DY021	March 2015	0.22	74.73	25.05	0.9	1.2	0.46	0.08	0.55
	DY030	May 2015	0.03	80.06	19.91	0.8	0.5	0.36	0.07	0.48
	DY034	August 2015	2.74	71.06	26.19	0.3	0.7	0.40	0.11	0.53
Site I	DY008	April 2014	0.02	66.05	33.93	1.5	NA	0.48	0.07	0.54
	DY021	March 2015	0.03	67.02	32.94	0.2	0.8	0.56	0.09	0.66
	DY030	May 2015	0.02	66.65	33.34	0.3	0.8	0.68	0.09	0.55
	DY034	August 2015	0.05	62.97	36.98	0.9	0.4	0.60	0.14	0.62
Site A	DY008	April 2014	0.00	35.49	64.51	1.6	NA	1.02	0.12	0.62
	DY021	March 2015	0.03	34.46	65.50	0.8	0.9	1.23	0.14	0.70
	DY030	May 2015	0.00	32.78	67.22	0.3	0.8	1.34	0.14	0.72
	DY034	August 2015	0.01	37.35	62.63	0.8	1.0	1.16	0.17	0.69

### Seasonal variation in sediment oxygen profiles

**Fig. 3:**
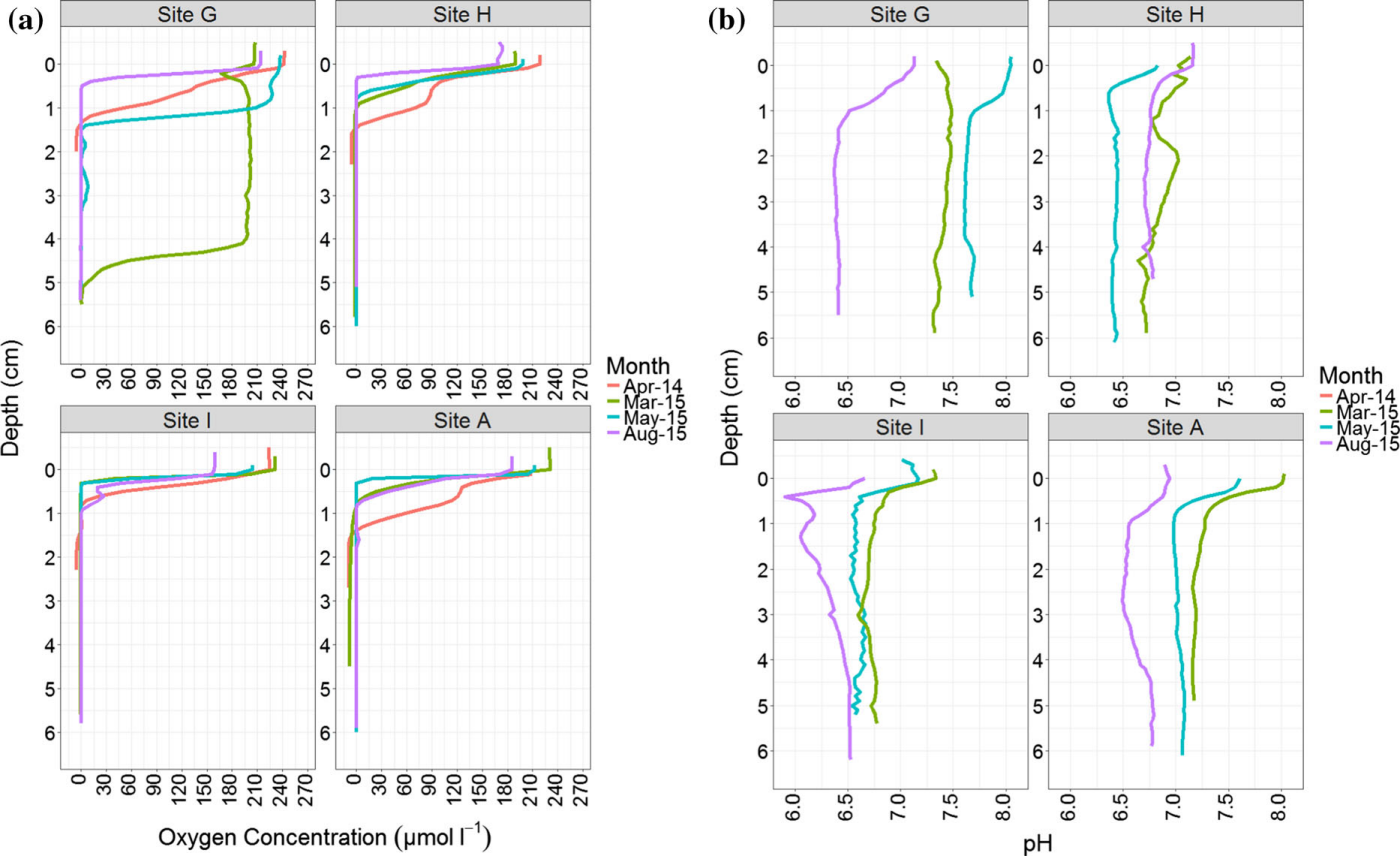
Temporal sediment profiles of **a** Oxygen concentration (umol/l) and **b** pH from four seasonal states: March 2015 (pre-spring bloom); April 2014 (pre-spring bloom); May 2015 (Bloom); August 2015 (post-bloom). No pH data was collected in April 2014

Overall, the oxygen microelectrode profiles show diffusional decreases with depth, with oxygen usually consumed in <2 cm depth from the sediment–water interface (Fig. 3a). Seasonal variability in oxygen concentration can be observed most strongly at Site G, the advective sand site. In the pre-spring bloom survey (March 2015) the OPD is at its deepest, reaching 5 cm, and there is minimal oxygen depletion from the surface throughout the top 4 cm of the profile. However, this deep OPD at Site G is not seen pre-spring bloom in the previous year (April 2014), possibly due to an earlier onset of the spring bloom (and accompanying water column stratification), as evident from the CTD data recorded throughout the cruise programme (Fig. 7b, Thompson et al. 2017). During the bloom (May 2015) the OPD shallowed reaching 1.4 cm, followed by a further shallowing to 0.5 cm post bloom (August 2015). Seasonally, at site A and site H the deepest OPDs were observed during the pre-spring bloom (April 2014) and at site I post-spring bloom (August 2015). The shallowest recorded OPD of 0.3 cm was observed at sites A and I during the bloom (May 2015) and post-bloom at Site H (August 2015).

### Seasonal variation in sediment pH profiles

The pH profiles show a decrease of up to a 0.5 pH unit in the upper 1 cm of the sediment (Fig. 3b). Similarly to oxygen, the gradient of this decrease and the point at which a minimum is reached can be variable. Below 1 cm the pH is remarkably constant with depth, in contrast to the North Sea profiles illustrated in Fig. 1. Site G again has the largest seasonal variability in pH signal (Fig. 3b), showing the largest range of pH sub-surface minima (1.2–4.3 cm). Observations are consistent between the pH and O_2_ profiles, exhibiting a relatively stable and overall higher pH profile (between 7 and 7.5) pre-spring bloom (March 2015) at Site G. This corresponds to a well flushed oxygenated sediment surface equilibrated with the overlying water column pH and thus consistent pH with increasing depth. This pH profile at Site G changes in shape over the seasons as the OPD shallows and hence oxidation of reduced species diffusing from below creates a discernible pH minima. The low pH at the sub-surface minima is maintained down profile to the maximum profiling depth. All the other sites show diffusive oxygen profiles with shallow OPDs and correspondingly the pH profiles exhibit pH decline in the surface of the sediment (<1 cm). At Site A and I pH values gradually decreases (Fig. 3b), both in the overlying water and in the sediment, as the seasons move from pre-spring bloom (March 2015) to bloom (May 2015) to post-bloom (August 2015). They also both show a pH increase at depth (>2 cm) not seen at the other sites. Contrastingly, Site H has very similar pH values when comparing pre-spring bloom (March 2015) to post-bloom (August 2015), with the lowest pH values during the bloom period (May 2016).

### Replication of measurements within a core

**Fig. 4:**
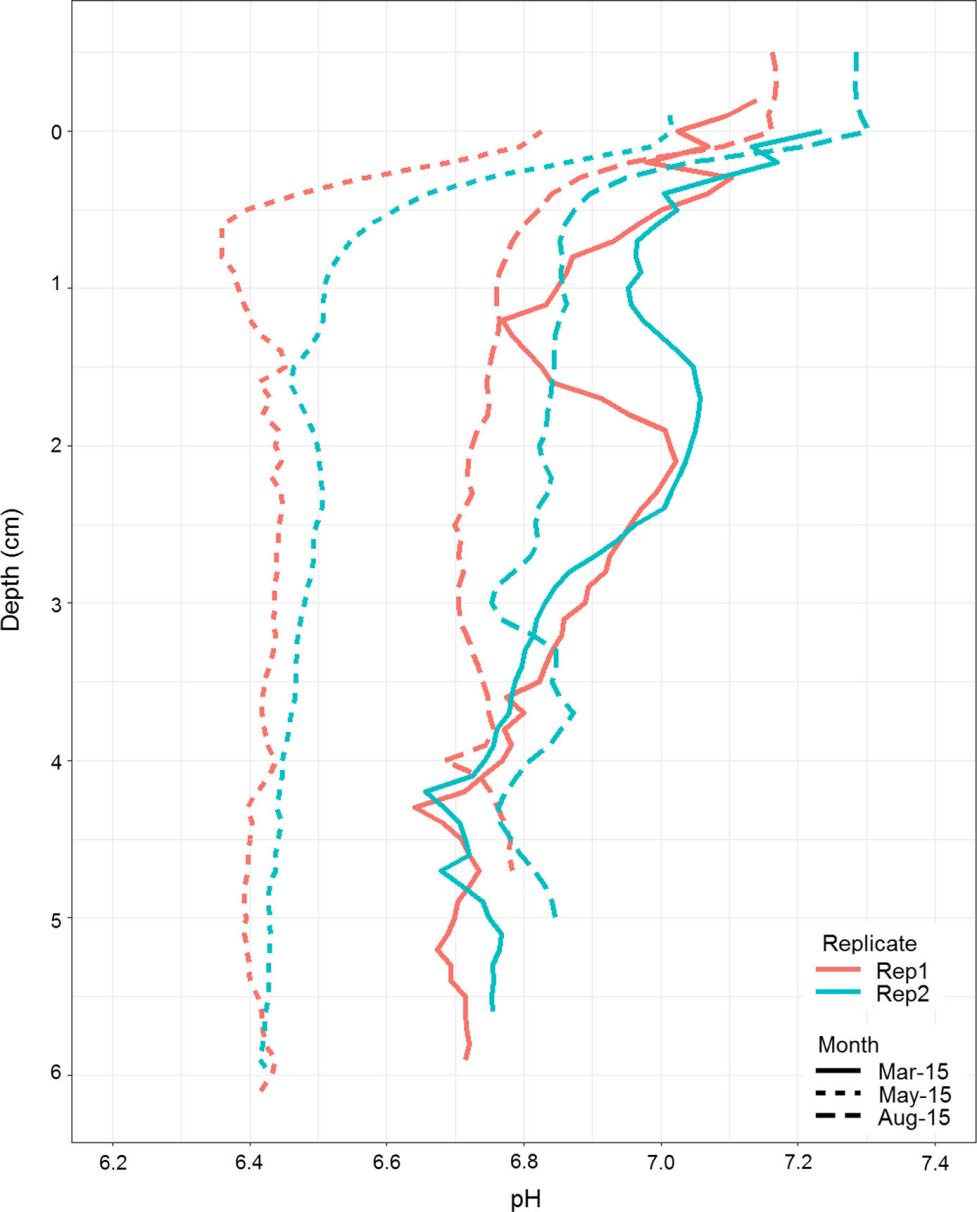
Replicate pH profiles from site H (*orange line Replicate 1, blue line Replicate 2*) from March, May and August 2015. No pH data was collected in April 2014. (Colour figure online)

To investigate the spatial heterogeneity of pH profiling within a subsampled core, replicate pH profiles were recorded immediately following the initial fresh ex situ profile (represented in Fig. 3b). These replicate profiles were sampled from within the same 10 cm diameter sub-core tube (<10 cm apart) and recording of the second profile commenced immediately following the first (~20–30 min after sub-sampling from the NIOZ). Results from site H are shown (Fig. 4), where the replicate profiles from within each core are shown in orange (Rep 1) and blue (Rep 2), with each cruise represented by different line types. Significant changes can be seen temporally across the profiles, as stated above, while pH maintains consistent features within the seasons. There is a general trend for the second replicate to have a slightly higher pH values throughout the profile, with the exception of March 2015. This slight increase in pH between replicates may be an artefact of the delay in profiling between the first and second replicate, allowing for the core temperature to rise slightly and in-bed properties to start breaking down, no longer closely reflecting in situ conditions. However, the agreement between replicates within the sub-core is greater than the differences observed between seasons (pre/post-bloom vs bloom; March/August 2015 vs May 2015). This confirms that the seasonal variation observed within the sites in Fig. 3b is not driven by pH discrepancies within a sub-core.

### Spatial characterisation of sediment types

**Fig. 5:**
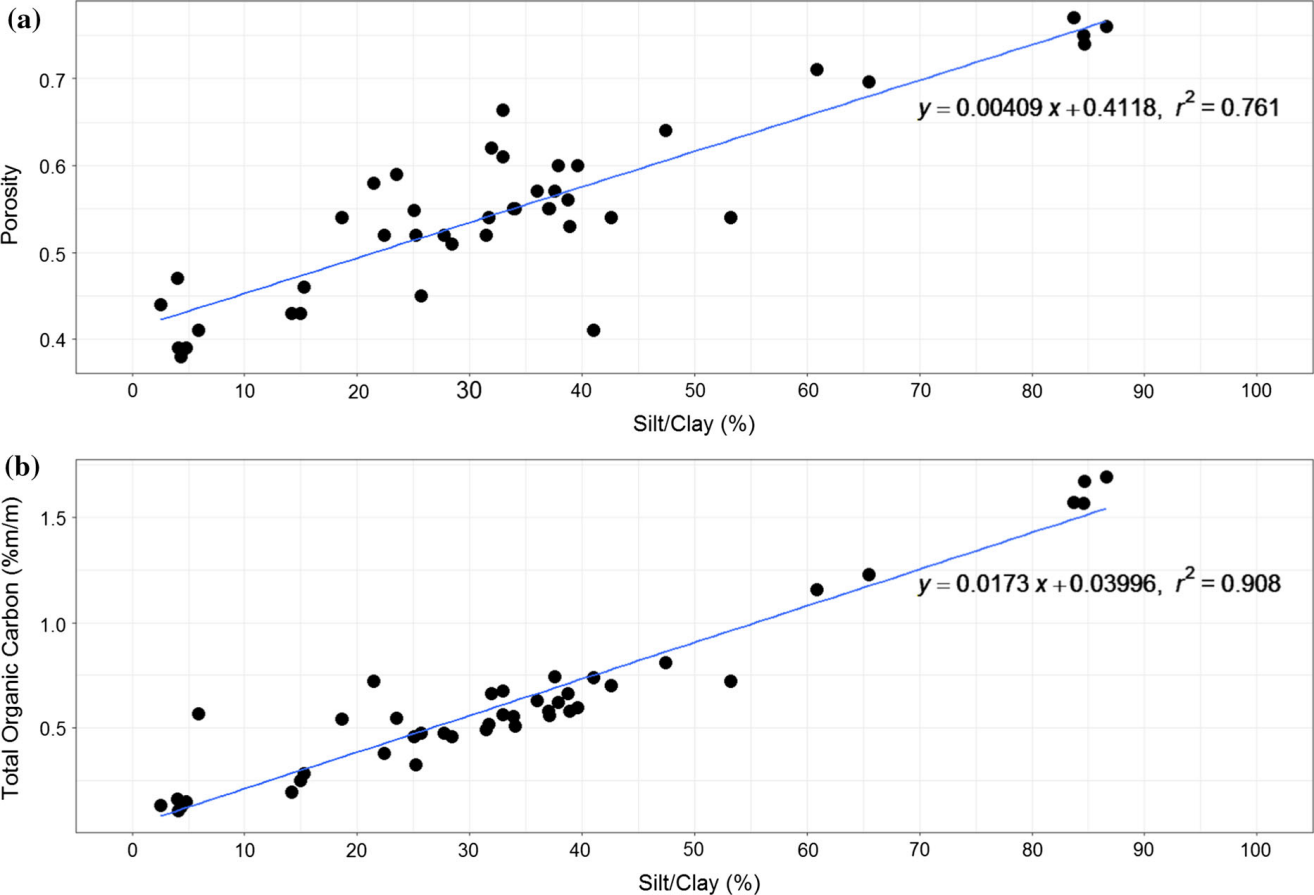
Percentage Silt/Clay vs **a** Porosity and **b** Total Organic Carbon (%m/m), with a linear model applied

The spatial survey carried out during the DY021 March 2015 survey (pre-spring bloom) successfully sampled 39 stations for all parameters, including pH and oxygen profiles. Combining this with the temporal stations creates an overall dataset of 43 stations across the Celtic Region. Sediment characterisation of the spatial stations confirms that the aim of the study to sample a wide range of sediment types was achieved. Silt/clay content by PSA of the 0-5 cm surficial sediment ranged from 2.55 to 86.61% (Fig. 5). Details of the specific PSA by spatial station ID can be found in the Thompson et al. Online Resource 3 (2017). The silt/clay content correlates strongly and significantly with total organic carbon (TOC) (r^2^ = 0. 908) and also shows a positive relationship with Porosity (r^2^ = 0. 761), relationships which are discussed in more details in terms of their statistical relevance and predictive power elsewhere (Diesing et al. 2017; Stephens and Diesing 2015).

### Spatial variability in sediment oxygen profiles

**Fig. 6:**
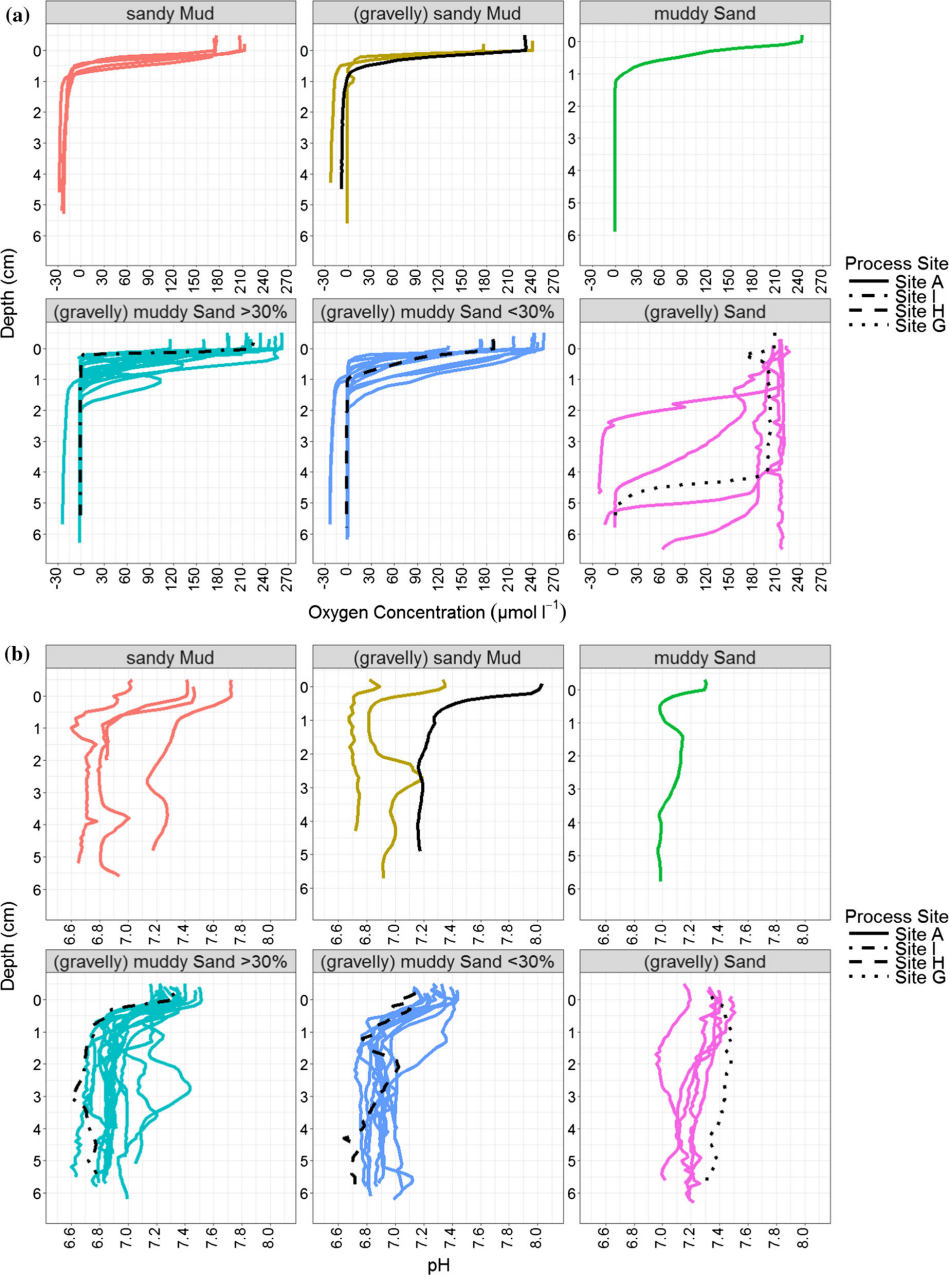
Oxygen and pH profiles of spatial survey stations and process sites, clustered and coloured by Folk and Ward textural classification groups (Folk 1954; Folk and Ward 1957)

Oxygen profiles from across the region, grouped by Folk and Ward sediment textural classification, are shown in Fig. 6a. The process site profiles from March 2015 are indicated by the black lines (line type discerns Site) and fall into different textural classifications as intended. The shallowing of the OPD occurs abruptly between the textural groups (gravelly) muddy Sand <30% and (gravelly) Sand, a result of the increasing silt/clay content of the muddy Sand. Within the (gravelly) Sand group, two stations (Spatial 45 and Spatial 16) have an OPD >6 cm which is not reached before profiling was aborted. Evidence of burrows can be seen in multiple profiles within the (gravelly) muddy Sand >30% group, indicated by the increasing of oxygen concentration at depth, before declining again to the oxygen minima. This is linked to bioirrigation or bioturbation processes of fauna in the surficial sediments.

### Spatial variability in sediment pH profiles

pH profiles were collected concurrently with the oxygen and are also grouped by textural classification in Fig. 6b. Steep pH gradients were recorded within the top few mm to cm in sandy Muds and (gravelly) sandy Muds, whilst the (gravelly) Sand sediments show pH at depth that is more consistent with surface sediment values. pH decline with depth therefore occurs at a shallower rate in sediment with a lower percentage silt/clay content, comparable to the deepening of the OPD. Overall, pH values and profile shape vary most significantly between (gravelly) Sand and the other classifications, reflecting the transition between diffusive and advective processes and associated redox changes. pH also varied between the sediment–water interface and deeper sediments. The maximum decrease in pH was from 8.02 (0 cm) to 7.16 (4.2 cm) at Site A, a difference of 0.86 pH units. In contrast, a pH decrease of 0.18 was measured at Site G, emphasising the clear differences between diffusive and advective sediments. The significance of these large changes in pH is even more striking considering the fact that pH is the free proton activity plotted on a logarithmic scale and as such a change of 1 unit de facto indicates an order of magnitude change in free proton concentration.

### Relationship between sediment type and both OPD and sub-surface pH minima

**Fig. 7:**
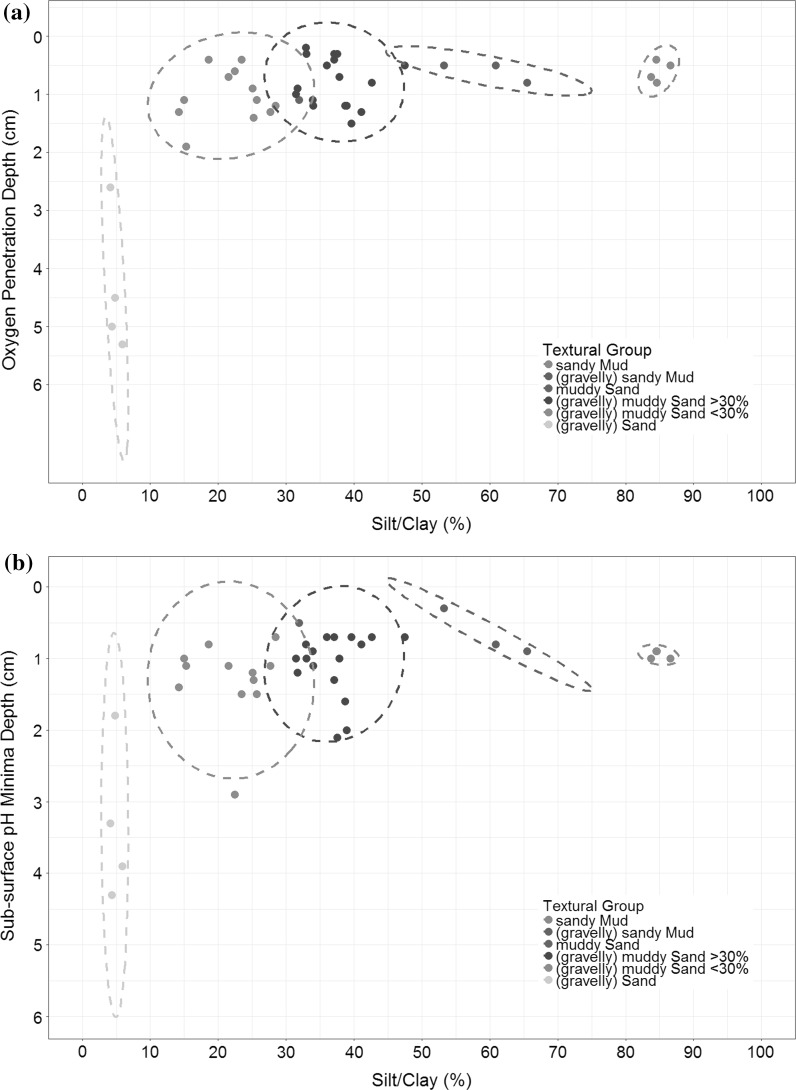
**a** OPD and **b** pH sub-surface minimum depth vs percentage silt/clay, clustered and coloured by Folk and Ward textural classification groups (Folk 1954; Folk and Ward 1957)

The relationship between OPD and percentage silt/clay (Fig. 7a) shows there is not a clear linear correlation, but rather a regressive one. It also becomes apparent that a breaking point at 10% fines content greatly increases the potential OPD and variability between depth of free oxygen found within different sediment textural groups. Above 10% fines content there is a general trend of shallowing OPD with increasing fines. It should be noted that there is no recorded OPD for Spatial 45 or 16 as the oxygen minima was not reached within the top 6 cm. These stations also had the lowest % fines content (2.55 and 4.02% respectively) and so are not represented on Fig. 7a. An elliptical model has been applied to the data, using the mean and spread of data to predict cluster groupings. This shows that the sand group stands out in its difference in OPD range in contrast to the similar OPDs across the other textural groups. This has been observed previously in sediment transects in the Channel and North Sea (Parker et al. 2011, 2012; Silburn et al., in prep). The relationship between pH and silt/clay (Fig. 7b) is congruent with that of OPD and has the same apparent breaking point at ≈10% fines content. However, the pH minimum sits lower in the sediment profile than the OPD. This is consistent with a shift towards sediment types which allow more reducing processes to occur at textural groups with >10% fines. The close link between OPD and pH minimum illustrates that the OPD is controlling the pH minimum, but potentially not the driving process that determines the pH profile, as it is likely this would be other H+ controlling sub-oxic processes.

**Fig. 8:**
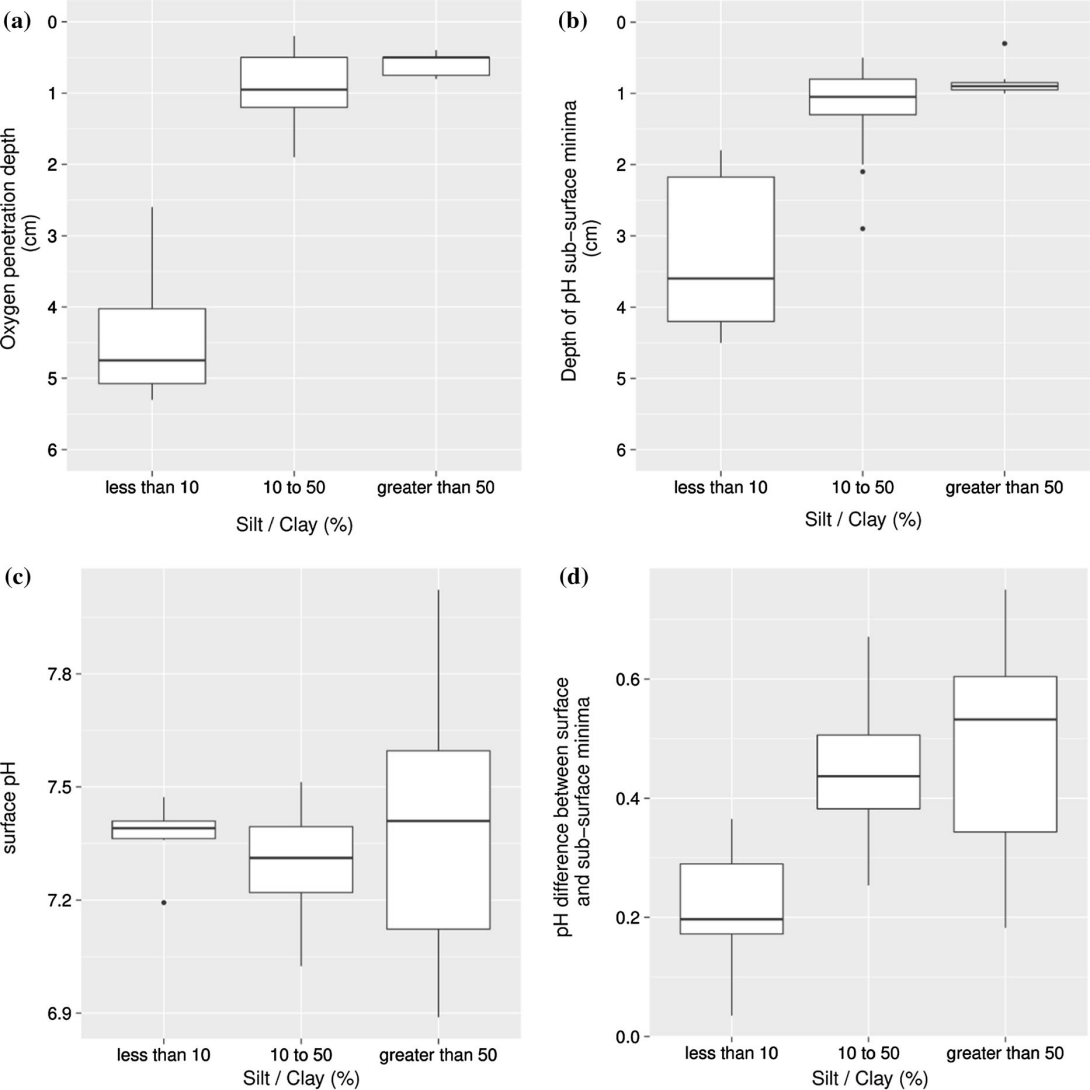
Box-and-whisker plots demonstrating the range of values of **a** oxygen penetration depth; **b** pH sub-surface minima depth; **c** near-bed pH (at 0 cm) and **d** difference between near-bed and sub-surface minima pH value, each categorised by silt/clay percentage content (<10%; 10–50%; >50%). *Horizontal lines* represent median values, box shows interquartile range and whiskers extend to the most extreme values not more than 1.5 times the interquartile range from the quartiles. Values beyond these limits are shown as individual points

In Fig. 8, the statistical significance of these correlations is further visualised. Box plots contrast silt/clay content, split into three categories (<10%; 10–50%; >50%), with OPD (Fig. 8a), pH sub-surface minima (Fig. 8b), near-bed pH (at 0 cm profiling depth) (Fig. 8c) and absolute difference between near-bed and sub-surface minima pH (Fig. 8d). T-tests were conducted on the data in Fig. 8 to assess the alternative hypothesis that the difference in means between different classes is not equal to zero, using a Welch two sample *t* test in R (R Core Team 2014). In Table 2, p-values are presented, which give the likelihood of the null hypothesis being true (that is, that the means are not different). A single asterisk denotes that this value is significant at 95% confidence (i.e., p < 0.05) and a double asterisk denotes significance at 99% confidence (i.e., p < 0.01). It is evident that there is a significant different between both the OPD and pH minima at <10% silt/clay in comparison to 10–50 and >50%, reaffirming the identification of a breaking point. There is also still a significant difference between the 10–50 and >50% silt/clay for both OPD and sub-surface minima, demonstrating the significant effect of increasing fines on both these variables. Surficial pH values are not significantly different across the ranges of silt/clay content, suggesting bottom waters across the survey area were consistent. However, the difference between the pH value at the surface and the pH value at the sub-surface minima were significantly different between sediment with <10% silt/clay and those with >10%.

**Table 2:** T-tests of silt/clay content (<10%, 10–50%, >50%) vs. OPD, pH sub-surface minima, near-bed pH or pH difference between surface and sub-surface minima value as generated in R

Oxygen penetration depth		
	10–50	>50
<10	0.01**	0.008**
10–50	–	0.007**

Depth of pH sub-surface minima		
	10–50	>50
<10	0.007**	0.004**
10–50	–	0.024*

Surface pH		
	10–50	>50
<10	0.17	0.88
10–50	–	0.58

pH difference between surface and sub-surface minima		
	10–50	>50
<10	0.004**	0.014*
10–50	–	0.61

*P*-values shown represent statistical probability that the null hypothesis is true. A single asterisk denotes significance at 95% confidence and a double asterisk denotes significance at 99% confidence

## Discussion

A range of sediment types were explored to identify how physical characteristics interact with the sediment oxygen and pH profiles. Microelectrode profiles were acquired on fresh cores only, achieving as near to in situ data as possible. However, possible impacts to the oxygen profiles include a slight shallowing of the OPD caused by the core recovery process (Glud 2008). This impact was managed by handling and subsampling cores with care, as well as profiling soon after (5–10 min) recovery to minimise core warming above bed temperatures.

Analysis of the spatial survey of pH sub-surface minimum and OPD has highlighted the significance of the transition between sediments of advective and diffusive properties in controlling the sediment conditions. In this study the boundary falls at ~10% fines. Other studies where this transition has been previously reported (Parker et al. 2011) have noted the boundary at approximately 5–8% fines in the North Sea. Given the low fines percentage and high sand content and porosity of these sediments, this is likely due to advective flow of porewater in the upper layers. This can be spatially and temporally variable due to heterogeneity of organic matter distribution or topographic (ripples) effects on pore-water flows. Once beyond the advective influence, a diffusional profile is established, governed by the processes discussed above. The variability in depth of the oxic zone and gradient of oxygen consumption is also related largely to temperature and hence rate of oxygen demand from within the sediment as limited by diffusional resupply from the overlying water column. Seasonal variability is possibly a results of organic detritus deposition on the sediment surface and either creating very high oxygen demand or effectively reducing/preventing advective processes. All process sites showed an increase in TOC content in the top 5 cm of sediment from May 2015 to March 2015, excluding Site H. This study also includes pH measurements and highlights how sediment characteristics and resulting regulation of predominant oxygen regime can influence or co-vary with pH levels and the depth of observed minima. The critical control on pH appears to be the switch from diffusive to advective substrates and the associated redox processes that follow accordingly. Clearly, other biological, chemical and physical factors (such as production or consumption of organic matter in the overlying water-column, or bed-shear stress through tides/waves or human impacts) also influence the fate of the organic matter once it arrives at the sediment water interface. These will also consequently influence its incorporation into the sediment to depth; the point at which metabolic controls begin to dominate. Furthermore sediment characteristics beyond those discussed, such as paleo-origin, chemical composition of the grain matrix and recent input as well as disturbance history all have to be considered. The profiles collected in this study demonstrate how variable, and frequently low, the measured pH is even within the upper sediment layers (<4–6 cm), with the observed pH varying between 0.18 and 0.87 pH units within a profile. It is most likely that this decrease is driven by oxidation of reduced species diffusing from lower down in the sediment column. The exception to this is where the full oxic layer is maintained to depth by advective porewater flow from the overlying water-column. Similar variances of between 0.5 and 1 unit and the presence of a sub-surface minima have been touched upon within the North Sea (Ostle et al. 2016) and other studies in San Diego Bay (Cai and Reimers 1993), within contaminated harbour sediment (Tankere-Muller et al. 2007) and 2D pH planar optode development experiments (Stahl et al. 2006). As in this study, these studies reported that the pH minima approximated in depth with the OPD and was probably linked to intensified oxidation of reduced metabolites (Fe, Mn, HS) release from sub-oxic or anaerobic degradation (Boudreau and Canfield 1988). Steeper gradients are likely caused by aerobic organic matter respiration or oxidation of reduced species (NH_4_^+^, Mn^2+^, Fe^2+^, S^−^,) while deeper down pH increases were observed in some sediments. These could be due to the isolated relocation of bottom water via macrofaunal bioirrigation or burrowing, or more generally due to anaerobic alkalinity generation (Thomas et al. 2008). This interpretation links well with Fe and Mn pore-water profiles taken at Site A, which are presented in Figs. 3 and 4 in Klar et al. (2017). The profiles show dissolved Fe release into pore waters at depth (4–6 cm) which is attributed to dissimilatory iron reduction (DIR) during the bacterial decomposition of organic matter. A decrease in dissolved Fe concentrations towards the surface is also observed, probably due to oxidation reactions within the oxic zone. Mn profiles show a reduction release close to the surface (0–2 cm) followed by a steady decrease with depth, showing a relationship between the sub-surface Mn maxima and the OPD. The resolution of the dissolved Mn profiles make it difficult to determine the location of Mn oxidation, but this will be above the reduction peak, within the oxic zone. Both Fe and Mn reactions will drive changes observed in the pH profiles, in particular the reoxidation of both metals within the oxic zone supplied by generation of dissolved forms of the metals at depth. Although the profiles reported in other studies exhibited similar pH sub-surface minima as observed in our study, there tended to be an area of pH increase below this oxidation zone, usually associated with Fe/Mn reduction. This feature is largely absent in the profiles presented here, despite the Fe and Mn reduction observed deeper in the sediments. Maintenance of this lower pH at depth may be related to S cycles or variability in CO_2_ dissociation reactions (partly pH dependant), however sulphide was below detection limit in the porewaters (Klar et al. 2017). A possible hypotheses to explain the absence of the secondary subsurface increase in pH is that the depth of the measured profiles was not deep enough to reach the corresponding biogeochemical zone. If the sediments are too well flushed, do not contain enough organic matter, or are too frequently disturbed, the clear zone of iron or magnesium reduction will be pushed deeper into the sediment. This is also evident from the profiles in Klar et al. (2017) discussed above, as they were able to profile to 12 cm and show the Fe reduction peak did not occur until ~6 cm. As additional data becomes available, the interpretation of the lack of variability of pH at depth will be improved. The variability in sediment surface pH is likely to be linked to water column pH changes, but also variance in oxic remineralisation of particulate organic matter which is deposited with patchy distribution during and post bloom. Previous studies have also found this inconsistency between pH porewater profiles and the pH of the bottom water immediately overlying the sediment (Rao et al. 2016; Shao et al. 2016). It is not consistent which site has the lowest or highest overlying pH and is likely influenced by fluxes from the sediment of carbon dioxide or variations in alkalinity generation or patch deposition of organic matter and fluff.

These results provide large scale, unique baseline measurements for shelf sea sediments against which to consider future impacts and changes in porewater pH, and consequently, near-bed acidification. This could include changes in pelagic and benthic proton gradients, the influence of benthic metabolism on near bottom acidification, or the impacts of climate change and human activities on benthic ecosystems. Currently pH observations in sediment porewaters and the water column adjacent to the sediment interface are sparse due to the methodological difficulties inherent in accessing and sampling this habitat. Previous studies have largely been dominated by experimental manipulative system, using incubated cores. These rigidly controlled environments are not reflective of true bed conditions and therefore pH profiles may be impacted by experimental artefacts. In addition, studies often focus on cohesive sediments, especially when profiling with microelectrodes, or have been restricted to one study site, sediment type or sampling period (Lohse et al. 1996; Rabouille et al. 2003; Taylor et al. 2015). This is due to the delicate nature of the glass microelectrodes and so understanding of pH profiles within coarser advective sediments is limited. The application of this method to a very wide range of sediment types, permeability ranges (including those dominated by diffusive and advective processes) and low to high organic carbon content is therefore novel and has resulted in the description of the associated complex pH climatology. Previous studies have also found that CO_2_ and pH values can vary widely across sediment types. Widdicombe et al. (2011) stated that, when compiling data from different marine environments, surficial sediment pH values ranged from between 6.5 to 8.2, which is broadly consistent with the findings reported in this study. They also state that although distributions of CO_2_ and pH are largely regulated by microbial redox reactions, the reworking of sediment by burrowing infauna can change the geochemical parameters of their surrounding sediment, thus resulting in a feedback between organisms and their environment (Widdicombe et al. 2011). There has also been discussion over the differing range of pH experienced in the top few cm when overlying water is exposed to increased levels of CO_2_ (Thistle et al. 2007). Since sedimentary pH is already known to vary widely relative to the pH of the overlying waters, faunal communities already experience, and are potentially adapted to, a wide range of pH within the top few cm (Widdicombe et al. 2009). Therefore, the implications of increasing water column acidification are possibly less extreme in comparison to the ranges and rates of change found in sediments. This study has demonstrated that the naturally occurring ranges that infauna living within the sediments must withstand are highly variable in terms of pH heterogeneity at both temporal and spatial scales. It also suggests the fauna living within the top layer or even in close proximity to the surface of shelf sea sediments may already have a pre-exposure or tolerance level to low pH values, a factor not generally taken into consideration during reporting of ocean acidification studies on benthic organisms. However, the interplay between water column organic matter supply, its degradation within sediments, and the resident macrofaunal community (in terms of controlling pH gradients, upon which ocean acidification will act) requires future examination. Past studies into sedimentary pH have focused on single sediment types or end members, such as muds versus sands or cohesive versus non-cohesive sediment (Queirós et al. 2014) rather than a range of sediment types. Other studies have been linked to method development (Cai and Reimers 1993; Stahl et al. 2006), explored environmental implications of sub-sea floor carbon dioxide release (Taylor et al. 2015), or focused on faunal implications of changing pH gradients within experimental scenarios (Dashfield et al. 2008; Widdicombe et al. 2009).

Future work looking at macrofaunal and microbial community assemblages may explain some of the controls affecting the OPD via bioirrigation and redox gradients, and setting the sub-oxic biogeochemical environment via bioturbation mechanisms redistributing organic matter. Another set of contributing factors yet to be explored in greater detail is that of human activities, such as trawling and dredging, and their impacts on O_2_ and pH gradients. Anything that perturbs sediment fabric, packing or organic matter mixing, and hence redox, will affect O_2_ and pH gradients within the sediments. In addition, seasonally or spatially variable organic matter supply could be driving pH (at least in the upper sediment layers, in the short term) as much as any water column effects in terms of long-term/spatial climatology.

## Conclusions and future work

This study has presented the largest number of sediment pH profiles, across the widest range of sediment types from a single study conducted in the Celtic Sea and comparable to wider European shelf sediment systems. It provides evidence of pH heterogeneity across sediment types not only on a spatial scale (both vertical into the sediment and horizontal between sampling sites), but also, within different sediment typology, a temporal basis as exemplified by the seasonal comparison of profiles from the same process sites visited multiple times. The later provide an insight into inter-seasonal changes in relation to plankton bloom cycling (water column production, bloom collapse and export of organic matter to the benthic system for storage or remineralisation).

The natural variability with depth observed confirms sediment pH is not static and that the depth of sub-surface pH minima, and other cycles within the sediment driving this, are likely to be highly changeable. Large pH differences within the profiles indicate the extent to which porewater proton concentrations are much more variable than those of the water column or near-bed layers. This is important because it has been assumed that small changes in the water column pH will have potentially adverse effects on benthic dwelling fauna (Widdicombe and Spicer 2008), however they may be better adapted to both lower pH and higher variability than previously thought. Under pH conditions, where increased particulate inorganic carbon dissolution occurs in the sediment, this may act as an additional source of pCO_2_. When released into the water column this may even be exacerbating ocean acidification driven changes in the overlying water column.

pH profiles studies of the scale presented here are rare, but must become more commonplace, if we are to fully understand the potential implications of climate change, ocean acidification and human impacts on the marine ecosystem. Until the system is fully explored and understood we can potentially expect modelling studies to incorrectly initialise or not to take account of existing present-day natural or anthropogenic variation in pH when extrapolating to future climate change scenarios.
